# Clinical risk factors and predictive score for the non-dipper profile in hypertensive patients: a case-control study

**DOI:** 10.1186/s40885-021-00180-4

**Published:** 2021-11-15

**Authors:** Chavalit Chotruangnapa, Titima Tansakun, Weranuj Roubsanthisuk

**Affiliations:** Division of Hypertension, Department of Medicine, Faculty of Medicine Siriraj Hospital, Mahidol University, 2 Wanglang Road, Siriraj, Bangkoknoi, Bangkok, 10700 Thailand

**Keywords:** Hypertension, Non-dippers, Risk factors, Blood pressure monitoring, Ambulatory

## Abstract

**Background:**

Night-time BP, especially non-dipper, is a stronger predictor of adverse cardiovascular outcomes. Ambulatory blood pressure monitoring (ABPM) is a gold standard for the detection of non-dippers but it often is unavailable and expensive. This study aims to determine clinical risk factors that predict non-dipper.

**Methods:**

An exploratory traditional case-control study, exclusive sampling of control was conducted from January 2013 to September 2018 to explore clinical risk factors associated with non-dippers in hypertensive patients. Subgroup analysis was performed in each treated and untreated hypertensive patient. The parsimonious predictive score for non-dippers was constructed.

**Results:**

The study included 208 hypertensive patients receiving 24 h ABPM. There were 104 dippers and 104 non-dippers. Significant clinical risk factors associated with non-dippers were the age of > 65 years, average office diastolic blood pressure (DBP), and fasting plasma glucose of > 5.6 mmol/L. Results of subgroup analysis showed that dyslipidemia, history of coronary artery disease, use of angiotensin-converting enzyme inhibitors (ACEIs) and direct vasodilators, average office DBP, and serum uric acid were associated with non-dippers in treated hypertensive patients, however, there were no risk factors associated with non-dippers in the untreated group. The predictive score for non-dippers in treated group included average office DBP, dyslipidemia, serum uric acid, male, calcium channel blockers and ACEIs use. The area under Receiver Operating Characteristic (AuROC) was 0.723. A cut-off point which was > 0.0701 and prevalence of non-dippers of 46%, this score had a sensitivity of 77.4%, specificity of 65.6%, positive predictive value (PPV) of 66.1%, and negative predictive value (NPV) of 79.6%. For untreated group, age, hemoglobin and body mass index were included in the predictive model. AuROC was 0.74. There was a sensitivity of 51.9%, specificity of 91.2%, PPV of 82.4%, and NPV of 70.5% at the cut-off point of > 0.357, and prevalence of 44%.

**Conclusion:**

There were several significant clinical risk factors associated with non-dippers in treated hypertensive patients. The predictive score might be useful for the detection of non-dippers; however, it cannot replace ABPM.

**Supplementary Information:**

The online version contains supplementary material available at 10.1186/s40885-021-00180-4.

## Introduction

Hypertension is a cause of death globally, accounting for 10.4 million death per year [1]. Uncontrolled hypertension is one of the most important risk factors for cardiovascular disease as well as increases morbidity and mortality [2, 3]. Blood pressure (BP) normally decreases during sleep by 10–20% of the average daytime BP in the normal population [4]. The nocturnal BP dipping can be calculated from the equation which was nocturnal BP dipping (%) = [(average daytime SBP – average nocturnal SBP)/ average daytime SBP]× 100. it is divided into four groups: extreme dippers (nocturnal BP dipping > 20%), dippers (nocturnal BP dipping 10–20%), non-dippers (nocturnal BP dipping 0 - < 10%) and reverse dipper (nocturnal BP dipping < 0%).

Ambulatory blood pressure monitoring (ABPM) is the best method for the detection of non-dippers and is a better predictor of hypertension-mediated organ damage (HMOD) than office BP [4, 5]. Previous studies found that night-time BP was a stronger predictor of adverse cardiovascular outcomes than daytime BP [5–8]. Non-dippers were related to sleep disturbance, obstructive sleep apnea, obesity, high salt intake, orthostatic hypotension, autonomic dysfunction, chronic kidney disease, diabetic neuropathy, metabolic syndrome, and old age [7–9].

However, ABPM is not widely available and has a high cost in Thailand. There were attempts to find the biomarkers for the prediction of non-dippers to replace ABPM. As the study of Gunebakmaz O et al. showed that a higher level of red cell distribution width (RDW) was related to non-dippers significantly [10] and Sunbul M et al. found that non-dippers had significantly higher neutrophil to lymphocyte ratio (NLR) and platelet to lymphocyte ratio (PLR) [11].

This study aimed to explore clinical risk factors and biomarkers that predicted non-dippers and to construct predictive scores for non-dippers.

## Materials and methods

### Study population and outcomes

This present study was a case-control study was conducted from a retrospective chart review in a hypertension clinic in Siriraj hospital from January 2013 to September 2018. The inclusion criteria were as follows: age was at least 18 years, underwent a 24 h ambulatory BP monitoring and have been diagnosed hypertension on one of the following criteria: 1) office systolic blood pressure (SBP) > 140 mmHg and/or diastolic blood pressure (DBP) > 90 mmHg or 2) home SBP > 135 mmHg and/or DBP > 85 mmHg or 3) ABPM: daytime mean SBP > 135 mmHg and/or DBP > 85 mmHg or night-time mean SBP > 120 mmHg and/or DBP > 70 mmHg or 24 h mean SBP > 130 mmHg and/or DBP > 80 mmHg or received anti-hypertensive medications. The exclusion criteria were as follows: pregnancy, peritoneal dialysis or hemodialysis, patients who received an ABPM for 24 h while admission. All patients underwent a 24 h ABPM for evaluation of dipping status. The patients who had at least 10% of nocturnal BP dipping were classified as dippers and the others were classified as non-dippers. In this study duration, there were 357 patients underwent a 24 h ABPM in Siriraj hospital. One hundred seventy-eight of these patients were non-dippers (Supplementary Table 1). The 104 cases were the patients who had non-dipping profiles and were randomly selected by computer. Patients with dippers were defined as controls. All controls were randomly selected by the exclusive sampling method at the end of the study from the same source of the cases. Hence, we included 208 patients (matching the 1:1 ratio of case and control) in this study. Because we mainly aimed to investigate the differences of RDW, NLR, and PLR, which might be new biomarkers for non-dippers as in previous studies [10, 11], between two groups. The previous study revealed that RDW of non-dippers was 14.1 + 1.33% while RDW in the other group was 13.58 + 0.89%. The total sample size estimation for each group was 104 with 90% power using the 5%-level two-sided test for detection of a mean difference of independence between two groups.

Data were collected from patient medical records at the last visit before ABPM included demographic data such as age, body mass index (BMI), current smoking, co-morbidities such as dyslipidemia, diabetes mellitus, ischemic stroke, cardiovascular disease, chronic kidney disease, renal artery stenosis, obstructive sleep apnea, thyroid disease, Cushing syndrome, primary aldosteronism, pheochromocytoma, and aortic disease. Diabetes mellitus was defined as fasting plasma glucose levels that were more than 7 mmol/L in multiple measurements, previously diagnosed diabetes mellitus, or the use of anti-diabetic medications. Dyslipidemia was defined as serum total cholesterol > 5.2 mmol/L, serum triglyceride > 1.7 mmol/L, low-density lipoprotein cholesterol > 3.4 mmol/L, previously diagnosed dyslipidemia, or use of lipid-lowering medications. Complete blood counts, which included total white blood cells, neutrophils, lymphocytes, hemoglobin, hematocrit, red cell distribution width (RDW), and platelets were obtained at the nearest time of performing 24 h ABMP. Neutrophil-lymphocyte ratio (NLR) and platelet-lymphocyte ratio (PLR) were calculated as the ratio of neutrophil count to lymphocyte count and as the ratio of platelet count to lymphocyte count. The renal function included BUN, creatinine, estimated glomerular filtration rate (eGFR), urine protein to creatinine ratio (UPCR), urine albumin to creatinine ratio (UACR). Stage of CKD, microalbuminuria and macroalbuminuria were classified as KDIGO 2012. Fasting plasma glucose, HbA1c, lipid profile and uric acid were collected, as well.

The study was approved by the Institutional Review Board of Faculty of Medicine Siriraj Hospital, Mahidol University (Certificate of Approval No. Si 108/2019).

### Statistical analysis

The demographic data of both cases and controls were presented and analyzed to compare clinical characteristics between both study groups. Continuous data were expressed as mean ± standard deviation or median and interquartile range, while categorical data are presented as counts and percentages. Chi-square test or Fisher exact test was used for comparison of categorical variables, while student t-test or Mann-Whitney U test was used to compare continuous variables depending on the distribution of data. Univariable logistic regression analysis was performed to determine which risk factors were associated with non-dipper status. Then, multivariable logistic regression was used for exploratory modeling to identify the independent risk factors for non-dippers. The selected parameters in this model consisted of the parameters that had the significant association of non-dipper from the univariable analysis. For the prevention of collinearity of the multivariable analysis model, we selected more significant collinear parameters. We also analyzed subgroups that were stratified by the treatment status of these patients. A *p*-value of less than 0.05 was considered statistically significant.

We used forward stepwise logistic regression to construct the 2 separate parsimonious models for non-dippers prediction of both treated and untreated hypertensive patients. Potential predictors were the variables with a *p*-value of less than 0.2 from the univariable logistic regression model and the important risk factors from the literature review. Receiver operating characteristic (ROC) curve analysis was performed to determine a cut-off score to predict the non-dipping status. The cut-off score was selected by using Youden index analysis. Model performances were presented with sensitivity, specificity, positive predictive value, and negative predictive value. Statistical analyses were performed using SPSS version 18.0 Chicago: SPSS Inc.

## Results

Of the 208 adult hypertensive patients who underwent 24 h, ABPM were enrolled in our study. All patients were divided into two groups: 104 dippers (42.3% of males, mean age was 53.5 ± 16.9 years old) and 104 non-dippers (31.7% of males, mean age was 63 ± 15 years old) The baseline characteristics of all participants in the two groups were shown in Table 1 (Supplementary Table 1 presented demographic data of our cohort). Non-dippers had significantly diabetes mellitus and dyslipidemia more than dippers. Non-dippers tended to use more numbers of anti-hypertensive medications but this difference did not reach statistical significance. The non-dipping group took calcium channel blockers (CCBs) and beta-blockers (BBs) as anti-hypertensive treatment more than another group. According to the definition of non-dipping status, night-time SBP was higher in non-dippers. In addition, patients with non-dippers had significantly lower all average office, mean of 24 h, daytime and night-time DBP as shown in Table 2.

**Table 1:** Demographic characteristics of all 208 patients

Characteristics	Total (***n*** = 208)	Dippers (***n*** = 104)	Non-dippers (***n*** = 104)	***P***-value
Age (years)	58.2 + 16.7	53.5 + 16.9	63.0 + 15.0	< 0.001
Male n (%)	77 (37)	44 (42.3)	33 (31.7)	0.114
Body weight (kg)	65.6 + 13.4	67.3 + 14.0	63.9 + 12.6	0.066
BMI (kg/m^2^)*	24.6 (21.7, 27.5)	24.7 (21.8, 27.6)	24.2 (21.5, 27.2)	0.523
Smoking n (%)	10 (4.8)	10 (9.6)	0 (0)	0.001
**Co-morbidities n (%)**				
Diabetes mellitus	47 (22.6)	13 (12.5)	34 (32.7)	< 0.001
Dyslipidemia	122 (58.7)	50 (48.1)	72 (69.2)	0.002
Obstructive sleep apnea	15 (7.2)	8 (7.7)	7 (6.7)	0.789
Ischemic stroke	12 (5.8)	2 (1.9)	10 (9.6)	0.017
Coronary artery disease	10 (4.8)	4 (3.8)	6 (5.8)	0.517
Heart failure	1 (0.5)	1 (1)	0 (0)	1.000
Chronic kidney disease	38 (18.3)	13 (12.5)	25 (24)	0.031
Number and type of anti-hypertensive medications n (%)				0.497
1	50 (24)	24 (23.1)	26 (25)	
2	39 (18.8)	22 (21.2)	17 (16.3)	
3	33 (15.9)	15 (14.4)	18 (17.3)	
4	19 (9.1)	6 (5.8)	13 (12.5)	
5	6 (2.9)	3 (2.9)	3 (2.9)	
Diuretics	28 (13.5)	15 (14.4)	13 (12.5)	0.685
CCBs	95 (45.7)	40 (38.5)	55 (52.9)	0.037
ACEIs	34 (16.3)	18 (17.3)	16 (15.4)	0.708
ARBs	66 (31.7)	30 (28.8)	36 (34.6)	0.371
Beta blockers	57 (27.4)	22 (21.2)	35 (33.7)	0.043
Peripheral alpha-1 blockers	27 (13)	11 (10.6)	16 (15.4)	0.302
Central acting alpha-2 agonists	9 (4.3)	4 (3.8)	5 (4.8)	1.000
Direct vasodilators	13 (6.2)	10 (9.6)	3 (2.9)	0.045
**Laboratory results**				
Hemoglobin (g/L)^a^	132 (120, 145)	134 (125, 148)	130 (117, 140)	0.006
Hematocrit (%)	40.6 + 4.8	41.6 + 4.5	39.7 + 4.8	0.005
RDW (%)^a^	13.6 (12.8, 14.6)	13.4 (12.7, 14.6)	13.7 (12.9, 14.6)	0.225
MCV (fl)^a^	88.5 (82.2, 91.9)	87.8 (80.9, 91.0)	89.1 (84.0, 92.0)	0.162
NLR^a^	1.80 (1.33, 2.44)	1.79 (1.29, 2.50)	1.85 (1.42, 2.36)	0.623
PLR^a^	123.25 (97.18, 161.71)	128.39 (98.91, 166.90)	120.95 (95.09, 158.13)	0.353
Fasting plasma glucose (mmol/L)^a^	5.5 (5.1, 6.3)	5.4 (4.9, 5.9)	5.8 (5.3, 6.4)	< 0.001
HbA1c (%)^a^	6.0 (5.6, 6.5)	6.0 (5.7, 6.4)	6.0 (5.6, 6.6)	0.655
Cholesterol (mmol/L)	4.72 + 0.91	4.84 + 0.89	4.61 + 0.92	0.060
Triglyceride (mmol/L)^a^	1.12 (0.88, 1.58)	1.15 (0.92, 1.71)	1.11 (0.87, 1.55)	0.495
HDL-cholesterol (mmol/L)	1.53 + 0.44	1.51 + 0.47	1.55 + 0.41	0.560
LDL-cholesterol (mmol/L)	2.61 + 0.84	2.72 + 0.86	2.50 + 0.80	0.054
Uric acid (mmol/L)	0.35 + 0.10	0.36 + 0.11	0.33 + 0.10	0.128
Abnormal proteinuria n (%)	55 (26.4)	20 (19.2)	35 (33.7)	0.346
Serum creatinine (μmol/L)^a^	76.02 (64.53, 97.24)	76.02 (63.65, 94.59)	76.02 (64.53, 103.43)	0.583
eGFR (ml/min/1.73 m^2^)	80.22 + 24.25	84.69 + 22.98	75.75 + 24.76	0.008

*BMI* body mass index, *CCBs* calcium channel blockers, *ACEIs* angiotensin converting enzyme inhibitors, *ARBs* angiotensin II receptor blockers, *RDW* red blood cell distribution width, *MCV* mean cell volume, *NLR* neutrophil-lymphocyte ratio, *PLR* platelet-lymphocyte ratio, *HbA1c* hemoglobin A1c, *HDL* high density lipoprotein, *LDL* low density lipoprotein, *eGFR* estimated glomerular filtration rate

^a^presented as median and 25th, 75th percentile

**Table 2:** Hemodynamic data of study population

Blood pressure	Total (*n* = 208)	Dippers (*n* = 104)	Non-dippers (*n* = 104)	*P*-value
Average office SBP (mmHg)	147.3 + 17.2	147.7 + 17.4	146.8 + 17.0	0.719
Average office DBP (mmHg)	83.1 + 11.9	86.0 + 12.2	80.2 + 10.8	< 0.001
24 h average SBP (mmHg)	128.6 + 14.7	128.3 + 14.1	128.9 + 15.4	0.782
24hourrs average DBP (mmHg)	71.5 + 10.9	73.6 + 11.9	69.4 + 9.4	0.005
Daytime SBP (mmHg)	132.2 + 15.5	134.6 + 14.9	129.7 + 15.8	0.022
Daytime DBP (mmHg)	73.9 + 12.1	77.8 + 12.8	70.0 + 9.9	< 0.001
Nighttime SBP (mmHg)	121.1 + 17.1	114.5 + 13.5	127.7 + 17.9	< 0.001
Nighttime DBP (mmHg)	66.4 + 11.2	64.5 + 11.3	68.2 + 10.8	0.017

*SBP* systolic blood pressure, *DBP* diastolic blood pressure

Determination of factors associated with non-dippers by using univariable logistic regression analysis was presented in Table 3. Exploratory modeling was analyzed by using multivariable logistic regression for exploration of independent risk factors of non-dippers as shown in Table 4. The independent risk factors for non-dippers were age of > 65 years (odds ratio 2.31, 95% confident interval (CI) 1.10–4.82), average office DBP (odds ratio 0.96, 95% CI 0.94–0.99) and impaired fasting plasma glucose (fasting plasma glucose was > 5.6 mmol/L) (odds ratio 2.15, 95% CI 1.04–4.47).

**Table 3:** Univariable logistic regression analysis for evaluation of the association between risk factors and non-dippers

Factors	Univariable analysis	
	Odds ratio (95% Confident Interval)	***P*** value
Age	1.04 (1.02–1.06)	< 0.001
Age > 65 years	3.33 (1.86–5.97)	< 0.001
Male	0.63 (0.36–1.12)	0.115
Bodyweight	0.98 (0.96–1.00)	0.069
BMI	0.98 (0.93–1.04)	0.069
Diabetes mellitus	3.40 (1.67–6.92)	0.001
Dyslipidemia	2.43 (1.38–4.28)	0.002
Obstructive sleep apnea	0.87 (0.30–2.48)	0.789
Ischemic stroke	5.43 (1.16–25.41)	0.032
Coronary artery disease	1.53 (0.42–5.59)	0.520
Chronic kidney disease	2.22 (1.06–4.62)	0.034
Diuretics	0.85 (0.38–1.88)	0.848
CCBs	1.80 (1.03–3.12)	0.037
ACEIs	0.87 (0.42–1.81)	0.708
ARBs	1.31 (0.73–2.35)	0.372
Beta-blockers	1.89 (1.02–3.52)	0.045
Peripheral alpha-1 blockers	1.54 (0.68–3.49)	0.305
Central acting alpha-2 agonists	1.26 (0.33–4.84)	0.734
Direct vasodilators	0.28 (0.08–1.05)	0.058
Use of > 1 anti-HT medications	1.39 (0.76–2.52)	0.287
Average office SBP	1.00 (0.98–1.01)	0.718
Average office DBP	0.96 (0.93–0.98)	0.001
Hemoglobin	0.78 (0.66–0.93)	0.004
Hematocrit	0.92 (0.86–0.98)	0.006
RDW	1.01 (0.86–1.19)	0.923
MCV	1.01 (0.98–1.04)	0.392
NLR	1.10 (0.88–1.37)	0.405
PLR	1.00 (1.00–1.01)	0.314
Fasting plasma glucose	1.02 (1.01–1.04)	0.006
Fasting plasma glucose > 5.6 mmol/L	2.74 (1.56–4.81)	< 0.001
HbA1c	1.40 (0.93–2.11)	0.112
Cholesterol	0.99 (0.99–1.000)	0.062
Triglyceride	1.00 (0.99–1.00)	0.269
HDL-cholesterol	1.01 (0.98–1.02)	0.558
LDL-cholesterol	0.99 (0.98–1.00)	0.055
Uric acid	0.87 (0.72–1.04)	0.129
Abnormal proteinuria	1.37 (0.71–2.64)	0.346
Serum creatinine	1.19 (0.61–2.32)	0.620
eGFR	0.98 (0.973–1.00)	0.009

*BMI* body mass index, *CCBs* calcium channel blockers, *ACEIs* angiotensin converting enzyme inhibitors, *ARBs* angiotensin II receptor blockers, *RDW* red blood cell distribution width, *MCV* mean cell volume, *NLR* neutrophil-lymphocyte ratio, *PLR* platelet-lymphocyte ratio, *HbA1c* hemoglobin A1c, *HDL* high density lipoprotein, *LDL* low density lipoprotein, *eGFR* estimated glomerular filtration rate

**Table 4:** Exploratory model by using multivariable logistic regression analysis for evaluation of the association between independent risk factors and non-dippers

Factors	Multivariable analysis	
	Odds ratio (95% Confident Interval)	***P*** value
Age > 65 years	2.31 (1.10–4.82)	0.026
Diabetes mellitus	1.10 (0.43–2.82)	0.846
Dyslipidemia	1.45 (0.71–2.94)	0.309
Ischemic stroke	3.85 (0.70–21.22)	0.122
CCBs	1.02 (0.49–2.09)	0.966
Beta blockers	1.07 (0.50–2.28)	0.863
Average office DBP	0.96 (0.94–0.99)	0.016
Hemoglobin	0.85 (0.70–1.03)	0.090
Fasting plasma glucose > 5.6 mmol/L	2.15 (1.04–4.47)	0.040
eGFR	1.00 (0.99–1.02)	0.721

*CCBs* calcium channel blockers, *DBP* diastolic blood pressure, *eGFR* estimated glomerular filtration rate

### Subgroup analysis by treatment status of hypertension

All dipping and non-dipping hypertensive patients were stratified into treated and untreated hypertensive groups. There were 147 patients in the treated hypertensive group. In this group, there were 70 and 77 patients with dipping and non-dipping status respectively. On the other hand, 61 patients were classified in the untreated hypertensive group. It consisted of 34 dippers and 27 non-dippers. The demographic data of both groups were presented in Supplementary Tables 2, 3, 4 and 5. For treated hypertensive group, dyslipidemia was positively associated with non-dippers (odds ratio 11.73, 95% CI 1.79–77.64) while the history of coronary artery disease (odds ratio 0.03, 95% CI 0.00–0.92), use of angiotensin-converting enzyme inhibitor (odds ratio 0.08, 95% CI 0.01–0.57) and direct vasodilators (odds ratio 0.04, 95% CI 0.00–0.74), average office DBP (odds ratio 0.89, 95% CI 0.82–0.96), as well as serum uric acid (odds ratio 0.62, 95% CI 0.41–0.93), had a negative association with non-dippers (Univariable analysis was shown in Supplementary Table 6 and multivariable analysis was shown in Table 5). Evening anti-hypertensive medications administration was the higher proportion in patients with coronary artery disease (11% versus 1.4% of patients without coronary artery disease; *p*-value = 0.017). Non-dippers who were treated by direct vasodilators were less frequently taken in the evening than dippers (16.7% of non-dippers versus 83.3% of dippers; *p*-value = 0.009). Losartan was used as a hypertensive treatment in 17% of non-dippers (13 patients) and 2% of dippers (1 patient) (*p*-value =0.001). However, there were not independent risk factors for non-dippers in untreated hypertensive groups (Univariable analysis was shown in Supplementary Table 7, and multivariable analysis was shown in Table 6).

**Table 5:** Exploratory model by using multivariable logistic regression analysis for evaluation of the association between independent risk factors and non-dippers in the treated hypertensive group

Factors	Multivariable analysis	
	Odds ratio (95% CI)	***P*** value
Age	1.011 (0.952–1.074)	0.721
Male	4.320 (0.611–30.564)	0.143
BMI	0.978 (0.845–1.133)	0.770
Diabetes mellitus	2.483 (0.347–17.764)	0.365
Dyslipidemia	11.773 (1.785–77.641)	0.010
Obstructive sleep apnea	1.505 (0.069–32.933)	0.795
Coronary artery disease	0.031 (0.001–0.924)	0.045
Diuretics	0.224 (0.026–1.938)	0.174
CCBs	1.788 (0.365–8.757)	0.473
ACEIs	0.076 (0.010–0.572)	0.012
ARBs	0.423 (0.081–2.197)	0.306
Beta-blockers	2.091 (0.486–9.003)	0.322
Peripheral alpha-1 blockers	1.446 (0.175–11.932)	0.732
Central acting alpha-2 agonists	0.737 (0.028–19.498)	0.855
Direct vasodilators	0.038 (0.002–0.743)	0.031
Evening drug administration	1.584 (0.395–6.355)	0.517
Average office SBP	1.052 (0.992–1.115)	0.092
Average office DBP	0.886 (0.818–0.959)	0.003
Hemoglobin	0.843 (0.507–1.399)	0.508
RDW	0.670 (0.401–1.120)	0.126
MCV	0.941 (0.842–1.052)	0.287
NLR	0.949 (0.386–2.331)	0.909
PLR	1.001 (0.995–1.007)	0.760
Fasting plasma glucose	0.972 (0.934–1.012)	0.168
Cholesterol	0.987 (0.910–1.069)	0.744
Triglyceride	0.996 (0.971–1.024)	0.818
HDL-cholesterol	0.980 (0.893–1.075)	0.672
LDL-cholesterol	1.014 (0.939–1.095)	0.720
Uric acid	0.615 (0.407–0.932)	0.022
Abnormal proteinuria	1.552 (0.306–7.871)	0.596
eGFR	0.999 (0.953–1.047)	0.966

*BMI* body mass index, *CCBs* calcium channel blockers, *ACEIs* angiotensin converting enzyme inhibitors, *ARBs* angiotensin II receptor blockers, *RDW* red blood cell distribution width, *MCV* mean cell volume, *NLR* neutrophil-lymphocyte ratio, *PLR* platelet-lymphocyte ratio, *HbA1c* hemoglobin A1c, *HDL* high density lipoprotein, *LDL* low density lipoprotein, *eGFR* estimated glomerular filtration rate

**Table 6:** Exploratory model by using multivariable logistic regression analysis for evaluation of the association between independent risk factors and non-dippers in the untreated hypertensive group

Factors	Multivariable analysis	
	Odds ratio (95% CI)	*P* value
Age	1.030 (0.917–1.157)	0.616
Male	1.200 (0.069–20.840)	0.901
BMI	1.055 (0.857–1.298)	0.614
Diabetes mellitus	0.022 (0.000–6.512)	0.189
Dyslipidemia	0.669 (0.019–23.167)	0.824
Average office SBP	0.943 (0.866–1.027)	0.175
Average office DBP	1.087 (0.954–1.239)	0.209
Hemoglobin	0.285 (0.073–1.117)	0.072
RDW	1.046 (0.358–3.056)	0.935
MCV	1.257 (0.944–1.675)	0.117
NLR	0.660 (0.153–2.846)	0.577
PLR	1.032 (0.991–1.074)	0.129
Fasting plasma glucose	1.036 (0.943–1.140)	0.459
Cholesterol	1.051 (0.688–1.606)	0.817
Triglyceride	0.999 (0.914–1.092)	0.985
HDL-cholesterol	0.894 (0.595–1.341)	0.588
LDL-cholesterol	0.932 (0.614–1.417)	0.744
eGFR	1.006 (0.896–1.130)	0.916
Abnormal proteinuria	2.216 (0.033–151.082)	0.712

*BMI* body mass index, *RDW* red blood cell distribution width, *MCV* mean cell volume, *NLR* neutrophil-lymphocyte ratio, *PLR* platelet-lymphocyte ratio, *HbA1c* hemoglobin A1c, *HDL* high density lipoprotein, *LDL* low density lipoprotein, *eGFR* estimated glomerular filtration rate

### The predictive score for non-dipper stratified by hypertensive treatment status

**Fig. 1:**
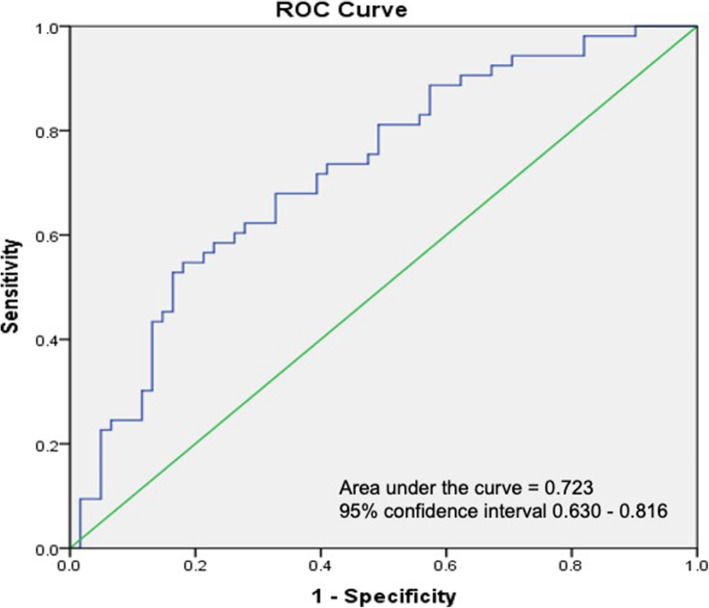
Receiver Operating Characteristic (ROC) analysis of predictive score model for non-dippers in treated hypertensive patients

The forward step-wise logistic regression analysis with potential predictive factors was performed to construct two predictive models. For treated hypertensive group, the predictive model for non-dippers was 7 - (0.081 x average office DBP) + (1.474 x dyslipidemia) - (0.297 x serum uric acid) + (1.031 x use of calcium channel blockers) - (0.986 x use of angiotensin-converting enzyme inhibitors) + (0.746 x male gender). Beta-coefficient of these prognostic factors and scoring for calculation this formula was presented in Table 7 and Supplementary Figure 1, respectively. The area under Receiver Operating Characteristic (AuROC) was 0.723 (Fig. 1). A cut-off point which was > 0.0701 and prevalence of non-dippers of 46%, this score had a sensitivity of 77.4%, specificity of 65.6%, positive predictive value (PPV) of 66.1%, and negative predictive value (NPV) of 79.6%.

**Table 7:** β co-efficient of the parameters of the predictive model for non-dippers by using forward stepwise logistic regression in the treated hypertensive group

Factors	β co-efficient	*P*-value*
Average office DBP	−0.081	0.001
Dyslipidemia	1.474	0.008
Serum uric acid	−0.297	0.046
Use of CCBs	1.031	0.059
Use of ACEIs	−0.986	0.087
Male	0.746	0.172
Constant	7.000	0.001

*CCBs* calcium channel blockers, *ACEIs* angiotensin-converting enzyme inhibitors

* *P*-value of less than 0.2 was considered statistically significant for this predictive model

**Fig. 2:**
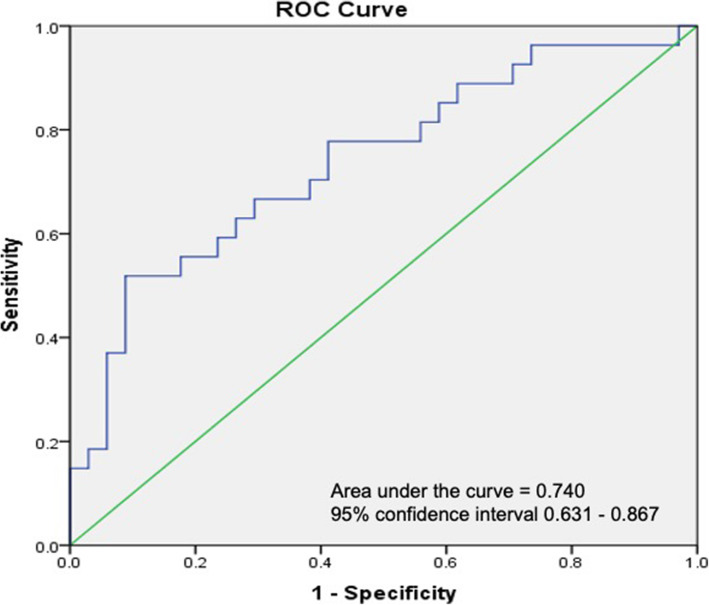
Receiver Operating Characteristic (ROC) analysis of predictive score model for non-dippers in untreated hypertensive patients

The predictive model for non-dippers in untreated hypertensive group was 5.443 + (0.030 x age) - (0.379 x hemoglobin) - (0.0933 x body mass index). Table 8 and Supplementary Figure 2 showed beta-coefficient of this prognostic factors and scoring for calculation this formula, respectively. AuROC was 0.74 (Fig. 2). There was sensitivity of 51.9%, specificity of 91.2%, PPV of 82.4% and NPV of 70.5% at cut-off point of > 0.357 and prevalence of 44%.

**Table 8:** β co-efficient of the parameters of the predictive model for non-dippers by using forward stepwise logistic regression in the untreated hypertensive group

Factors	β co-efficient	***P***-value*
Age	0.030	0.114
Body mass index	−0.093	0.146
Hemoglobin	−0.379	0.064
Constant	5.443	0.120

* *P*-value of less than 0.2 was considered statistically significant for this predictive model

## Discussion

Our study’s finding revealed that the clinical risk factors associated with non-dippers and the mathematical model for the predictive score of hypertensive patients with non-dipping status. The independently associated risk factors for non-dipping status in our study were the elderly (age of > 65 years) and impaired fasting plasma glucose (fasting plasma glucose was > 5.6 mmol/L). In contrast, average office DBP was negatively associated with non-dippers because of the high prevalence of isolated systolic hypertension. These findings were in accordance with the results of the study of Alejandro de la Sierra et al. that analyzed factors associated with blunted night-time BP dipping by using data from the Spanish Society of Hypertension Ambulatory Blood Pressure Monitoring Registry which obtained 24 h ABPM data from 42,947 hypertensive patients. They showed that advanced age, obesity, DM, and overt cardiovascular and renal disease were associated with non-dippers [12]. The elderly has high diurnal BP variability because of arterial stiffness and autonomic failure. After 60–70 years of age, 24 h SBP predominantly increases while 24 h DBP slightly decreases so isolated systolic hypertension is prevalent in elderly people [8]. This could be the reason why low average office DBP related to non-dippers. Hyperglycemia, including impaired fasting plasma glucose and DM, was found to be associated with non-dippers because hyperinsulinism in an insulin-resistant state causes sodium retention and alteration of arterial structure and function. Furthermore, poor glycemic control will result in autonomic dysregulation [13]. Although some evidence supported that hypertensive patients with non-dippers had increase platelet activation and a high inflammatory state [14], the inflammatory biomarkers, which were RDW, MCV, NLR, and PLR, in our study did not significantly associate with non-dipping status. Our results contrasted with Gunebakmaz O et al. and Sunbul M et al.’s ones because of the different study populations’ characteristics. The study of Gunebakmz O et al. included age and gender matching between the subjects with and without hypertension [10]. The inclusion criteria in the study of Gunebakmz O et al. and Sunbul M et al. were similar but only subjects with hypertension were included in the study of Sunbul M et al. [11]. Both previous studies excluded the subjects with heart disease (e.g. coronary artery disease, chronic heart failure), cerebrovascular disease, hematologic disorders (e.g. anemia, thrombocytopenia), malignancy, renal or hepatic dysfunction, secondary hypertension, autoimmune disease and systemic inflammation. Although the inclusion criteria of our study were similar to both previous studies, we did not exclude patients with cardiovascular disease, stroke, kidney disease and hematologic problem (for example anemia). Even though daytime SBP in non-dippers was lower than dippers, nighttime blood pressure phenotype still predicted adverse cardiovascular outcomes and was not dependent on daytime blood pressure. This hypothesis was supported by the study of Hong-Qi Fan, et al. which showed that isolated nocturnal hypertension predicted cardiovascular outcome [15]. We considered the effect of antihypertensive medication on dipping status; hence we analyzed the association of risk factors and non-dippers stratified by treatment status of hypertensive patients. For the treated hypertensive group, dyslipidemia was associated with non-dippers because dyslipidemia is an atherosclerotic risk factor of cardiovascular disease and is associated with metabolic syndrome. A study by Sipping Dai, et al. showed that non-dipping hypertensive patients with dyslipidemia were associated with cardiovascular disease [16]. Nevertheless, history of coronary artery disease, use of angiotensin-converting enzyme inhibitor and direct vasodilators as well as serum uric acid had a negative relation with non-dippers. Because of the high proportion of evening administration of antihypertensive medications in patients with coronary artery disease and the ones who took direct vasodilators, we supposed that evening administration of direct vasodilators was able to decrease night-time BP. It was supported by previous studies such as the Hygia Chronotherapy trial of Hermida RC et al. [17] demonstrated the efficacy of blood pressure lowering drugs at bedtime on improvement of blood pressure control and dipping status. But the exact mechanism of using direct vasodilators themselves negatively related to non-dippers was unknown. The evidence of the efficacy of direct vasodilators on dipping status and nocturnal blood pressure control is also limited. We suggested that further research to determine which blood pressure lowering drugs should be taken in the evening for the treatment of non-dipping status and the mechanistic explanation of those drugs are required. Some studies revealed that serum uric acid was associated with non-dipping status [18, 19]. Giallauria F et al. and Turak O et al.’s ones demonstrated the positive association of serum uric acid and non-dipping circadian pattern in newly diagnosed untreated hypertensive patients without treatments affected uric acid metabolism (such as allopurinol) in order to control the confounders [18, 19]. Serum uric acid in our study was negatively associated with non-dippers because it might be modified by the uricosuric effect of losartan, which was taken in non-dippers more than dippers. Uric acid lowering property of losartan had been shown in several studies [20–22]. So losartan was the important confounder that might cause the negative association between serum uric acid and non-dipping status in our study.

It is well known that blood pressure from ABPM is a better predictor for cardiovascular and renal disease than office BP. ABPM can provide nocturnal blood pressure, which is correlated with adverse cardiovascular and renal outcomes. Several hypertension guidelines including Thai hypertension guideline suggest considering ABPM as one method of out of office BP measurement. In Thailand, 24 h ABPM is currently the only method to evaluate night-time BP but it is not widely available due to the high cost of ABPM devices. Current evidence supports that non-dippers are related to hypertension-mediated organ damage [23–26]. In addition, extreme dippers also result in myocardial and brain ischemia at night time [27, 28]. Therefore, precise diagnosis of non-dippers in hypertensive patients will specifically lead to improvement of blood pressure control and reduction of adverse cardiovascular events. We proposed two new mathematical models for the prediction of non-dipping status by using clinical data and basic investigation in the treated and untreated hypertensive group. By using forward stepwise multivariable logistic regression for constriction of predictive score, the usages of calcium channel blockers (CCBs) and angiotensin-converting enzyme inhibitors (ACEIs) reached the statistical significance that we had decided. In addition, ACEIs were effective in decreasing nocturnal blood pressure, especially when given at bedtime [29]. The pathophysiology of non-dipping is complex. It may involve sodium retention, activation of sympathetic nervous system and renin-angiotensin-aldosterone system [30–32]. Dihydropyridine calcium channel blockers are the mainstay of the treatment of hypertension. The studies of dihydropyridine calcium channel blockers treatment and non-dipping hypertensive patients had mixed results depending on pharmacokinetics, timing of administration and patients’ profiles (e.g. dipping status, resistant hypertension, etc.) [33–35]. Luo Y et al.’s study demonstrated that 24 h BP profiles were normalized by taking amlodipine at the evening time [35]. In contrast with this study, all treated hypertensive patients in our study took CCBs in the morning. We did not know the exact mechanism why ACEIs or CCBs attenuate non-dipping status and further investigations to clarify these associations are required.

The performance of our predictive models had fair accuracy. There are no standard cut-off points so we used the Youden index statistic for the selection of cut-off points. Nevertheless, the overall accuracy of the predictive model was not as good as ABPM. It might result from a small sample size. Hence, our predictive model cannot replace ABPM. Further study with a larger sample size is needed to improve the accuracy of the predictive model and perform external validity.

### Strengths and limitations

This study showed the risk factors which were independent predictors of non-dipping status in hypertensive patients and provided the first parsimonious predictive model for Thai hypertensive patients. These models can be applied in both hypertensive patients with and without treatment by anti-hypertensive medications because separate analysis and modeling were performed according to the treatment status.

There were some limitations in this study. First, missing data and miss-classification bias was the problem in the retrospective study. The interval from data collection of risk factors to ABPM application varied in each participant because of different follow-up intervals and frequency of individual laboratory testing. Thus, it may affect the association between risk factors and outcome. Second, our predictive models need external validation. We have planned to use these predictive models in real-world practice and to re-analyze their accuracy as well as to improve their precision in the next step. Third, there is not enough sample size to perform external validation in this study.

## Conclusion

There were several significant clinical risk factors associated with non-dippers in treated hypertensive patients. The two predictive models stratified by treatment status of hypertension might be useful for the detection of non-dippers in real-world clinical practice, particularly in primary and secondary care hospitals. However, it cannot replace ABPM.

## Supplementary Information

**Additional file 1: Supplementary Table 1.** Baseline characteristics of the cohort. **Supplementary Table 2.** Demographic characteristics of treated hypertensive patients. **Supplementary Table 3.** Hemodynamic data of treated hypertensive groups. **Supplementary Table 4.** Demographic characteristics of untreated hypertensive patients. **Supplementary Table 5.** Hemodynamic data of untreated hypertensive groups. **Supplementary Table 6.** Univariable logistic regression analysis for evaluation of the association between risk factors and non-dippers in the treated hypertensive group. **Supplementary Table 7.** Univariable logistic regression analysis for evaluation of the association between risk factors and non-dippers in the untreated hypertensive group.

**Additional file 2: Supplementary Figure 1.** Predictive score model for non-dippers in treated hypertensive patients.

**Additional file 3: Supplementary Figure 2.** Predictive score model for non-dippers in untreated hypertensive patients.

## Data Availability

Not applicable.
