# Supplementary material for: Clinical risk factors and predictive score for the non-dipper profile in hypertensive patients: a case-control study
Source: Clin Hypertens. 2021 Nov 15;27:22. doi: 10.1186/s40885-021-00180-4 (PMC8591836; doi:10.1186/s40885-021-00180-4)
Supplement: Supplementary file 1 — Additional file 1: Supplementary Table 1. Baseline characteristics of the cohort. Supplementary Table 2. Demographic characteristics of treated hypertensive patients. Supplementary Table 3. Hemodynamic data of treated hypertensive groups. Supplementary Table 4. Demographic characteristics of untreated hypertensive patients. Supplementary Table 5. Hemodynamic data of untreated hypertensive groups. Supplementary Table 6. Univariable logistic regression analysis for evaluation of the association between risk factors and non-dippers in the treated hypertensive group. Supplementary Table 7. Univariable logistic regression analysis for evaluation of the association between risk factors and non-dippers in the untreated hypertensive group. [file 40885_2021_180_MOESM1_ESM.docx]

**Supplementary Table 1:** **Baseline characteristics of the cohort.**

| **Characteristics** | **Total**  **(n = 357)** | **Dipper**  **(n = 179)** | **Non-dipper**  **(n = 178)** | **P-value** |
| --- | --- | --- | --- | --- |
| Age (years) | 55.69 + 18.59 | 49.30 + 18.28 | 62.11 + 16.61 | < 0.001 |
| Male n (%) | 139 (38.9) | 82 (45.8) | 57 (32) | 0.008 |
| Body weight (kg)* | 65  (55.4, 74) | 68  (57.9, 77.1) | 63  (54.5, 71.2) | 0.001 |
| Body mass index (kg/m^2^)* | 24.61  (21.48, 27.83) | 24.88  (21.85, 28.10) | 24.08  (20.64, 27.28) | 0.076 |
| Obstructive sleep apnea n (%) | 26 (7.3) | 14 (7.8) | 12 (6.7) | 0.695 |
| Diabetes mellitus n (%) | 70 (19.6) | 19 (10.6) | 51 (28.7) | < 0.001 |
| Dyslipidemia n (%) | 187 (52.4) | 72 (40.2) | 115 (64.6) | < 0.001 |
| Ischemic stroke n (%) | 26 (7.3) | 7 (3.9) | 19 (10.7) | 0.014 |
| Coronary artery disease n (%) | 21 (5.9) | 6 (3.4) | 15 (8.4) | 0.042 |
| Heart failure n (%) | 3 (0.8) | 1 (0.6) | 2 (1.1) | 0.623 |
| Chronic kidney disease n (%)^†^ | 64/336 (19) | 15/163 (9.2) | 49/173 (28.3) | < 0.001 |
| **Number and type of anti-hypertensive drugs n(%)** | |  |  | 0.026 |
| 1 | 82 (23) | 40 (22.3) | 42 (23.6) |  |
| 2 | 64 (17.9) | 35 (19.6) | 29 (16.3) |  |
| 3 | 48 (13.4) | 18 (10.1) | 30 (16.9) |  |
| 4 | 29 (8.1) | 9 (5) | 20 (11.2) |  |
| 5 | 11 (3.1) | 4 (2.2) | 7 (3.9) |  |
| 6 | 1 (0.3) | 0 (0) | 1 (0.6) |  |
| Diuretics | 43 (12) | 20 (11.2) | 23 (12.9) | 0.612 |
| CCBs | 155 (43.4) | 66 (36.9) | 89 (50) | 0.012 |
| ACEIs | 50 (14) | 25 (14) | 25 (14) | 0.983 |
| ARBs | 95 (26.6) | 38 (21.2) | 57 (32) | 0.021 |
| Beta-blockers | 87 (24.4) | 31 (17.3) | 56 (31.5) | 0.002 |
| Peripheral alpha-1 blockers | 53 (14.8) | 16 (8.9) | 37 (20.8) | 0.002 |
| Central acting alpha-2 agonists | 15 (4.2) | 8 (4.5) | 7 (3.9) | 0.800 |
| Direct vasodilators | 27 (7.6) | 14 (7.8) | 13 (7.3) | 0.853 |
| Average office SBP (mmHg) | 146.61 + 21.93 | 145.74 + 25.39 | 147.47 + 17.87 | 0.457 |
| Average office DBP (mmHg) | 82.51 + 14.26 | 84.24 + 16.71 | 80.78 + 11.08 | 0.022 |
| 24hours average SBP (mmHg) | 129.31 + 15.08 | 128.51 + 13.93 | 130.12 + 16.15 | 0.314 |
| 24hours average DBP (mmHg) | 72.40 + 11.07 | 73.66 + 10.76 | 71.13 + 11.27 | 0.031 |
| Daytime SBP (mmHg) | 132.69 + 15.37 | 134.70 + 14.50 | 130.68 + 15.98 | 0.013 |
| Daytime DBP (mmHg) | 74.80 + 11.91 | 77.78 + 11.40 | 71.83 + 11.70 | < 0.001 |
| Nighttime SBP (mmHg) | 121.76 + 18.06 | 114.35 + 13.14 | 129.17 + 19.26 | < 0.001 |
| Nighttime DBP (mmHg) | 67.06 + 11.51 | 64.33 + 10.34 | 69.80 + 11.98 | < 0.001 |
| Hemoglobin (g/L)* | 131  (119, 144) | 134  (125, 147) | 129  (116, 140) | < 0.001 |
| Hematocrit (%) | 40.44 + 4.78 | 41.62 + 4.38 | 39.38 + 4.89 | < 0.001 |
| RDW (%)* | 13.45  (12.80, 14.50) | 13.30  (12.75, 14.10) | 13.70  (12.85, 14.60) | 0.023 |
| MCV (fl)* | 88.00  (82.60, 92.00) | 87.60  (82.20, 91.10) | 89.00  (83.85, 92.05) | 0.287 |
| NLR* | 1.87  (1.39, 2.54) | 1.80  (1.31, 2.64) | 1.94  (1.45, 2.50) | 0.417 |
| PLR* | 127.06  (98.82, 172.93) | 132.28  (100.79, 172.47) | 121.67  (96.18, 174.67) | 0.326 |
| Fasting plasma glucose (mmol/L)* | 5.5  (5.1, 6.1) | 5.3  (5, 5.7) | 5.7  (5.2, 6.3) | < 0.001 |
| Hemoglobin A1c (%)* | 5.9  (5.6, 6.4) | 5.9  (5.5, 6.3) | 6.0  (5.6, 6.6) | 0.177 |
| Cholesterol (mmol/L) | 4.65 + 0.95 | 4.77 + 0.93 | 4.54 + 0.96 | 0.035 |
| Triglyceride (mmol/L) | 1.30 + 0.64 | 1.31 + 0.67 | 1.29 + 0.60 | 0.975 |
| HDL-cholesterol (mmol/L) | 1.50 + 0.44 | 1.46 + 0.44 | 1.53 + 0.43 | 0.164 |
| LDL-cholesterol (mmol/L) | 2.60 + 0.81 | 2.73 + 0.83 | 2.48 + 0.77 | 0.006 |
| Serum uric acid (mmol/L) | 0.34 + 0.10 | 0.35 + 0.10 | 0.34 + 0.10 | 0.231 |
| Abnormal proteinuria n(%) | 64 (17.9) | 26 (14.5) | 38 (21.3) | 0.320 |
| Serum creatinine (μmol/L) | 89.28 + 52.16 | 80.44 + 34.48 | 97.24 + 63.65 | 0.014 |
| eGFR (ml/min/1.73 m^2^) | 81.18+27.12 | 89.50+24.42 | 73.34+27.27 | < 0.001 |

CCBs = calcium channel blockers; ACEIs = angiotensin converting enzyme inhibitors; ARBs = angiotensin II receptor blockers; SBP = systolic blood pressure; DBP = diastolic blood pressure; RDW = red blood cell distribution width; MCV = mean cell volume; NLR = neutrophil-lymphocyte ratio; PLR = platelet-lymphocyte ratio; HbA1c = hemoglobin A1c; HDL = high density lipoprotein; LDL = low density lipoprotein, eGFR = estimated glomerular filtration rate

*presented as median and 25^th^, 75^th^ percentile

^†^The number of case/ the number of total study population without missing data of chronic kidney disease

**Supplementary Table 2: Demographic characteristics of treated hypertensive patients.**

| **Characteristics** | **Total**  **(n = 147)** | **Dippers**  **(n = 70)** | **Non-dippers**  **(n = 77)** | **P-value** |
| --- | --- | --- | --- | --- |
| Age (years) | 61.0 + 15.9 | 56.4 + 15.9 | 65.2 + 14.8 | <0.001 |
| Male n (%) | 60 (40.8) | 33 (47.1) | 27 (35.1) | 0.179 |
| Body weight (kg) | 67.0 + 13.4 | 68.4 + 13.5 | 65.8 + 13.2 | 0.240 |
| BMI (kg/m^2^)* | 25.4 (22.4, 28.0) | 25.5 (22.2, 28.0) | 25.3 (22.5, 28.0) | 0.921 |
| Smoking n (%) | 9 (6.1) | 9 (12.9) | 0 (0) | 0.001 |
| **Co-morbidities n (%)** |  |  |  |  |
| Diabetes mellitus | 42 (28.6) | 11 (15.7) | 31 (40.3) | 0.001 |
| Dyslipidemia | 105 (71.4) | 41 (58.6) | 64 (83.1) | 0.002 |
| Obstructive sleep apnea | 14 (9.5) | 7 (10.0) | 7 (9.1) | 1.000 |
| Ischemic stroke | 8 (5.4) | 2 (2.9) | 6 (7.8) | 0.280 |
| Coronary artery disease | 9 (6.1) | 3 (4.3) | 6 (7.8) | 0.499 |
| Chronic kidney disease | 34 (23.1) | 13 (18.6) | 21 (27.3) | 0.243 |
| **Number and type of anti-hypertensive drugs n (%)** | |  |  | 0.514 |
| 1 | 50 (34.0) | 24 (34.3) | 26 (33.8) |  |
| 2 | 39 (26.5) | 22 (31.4) | 17 (22.1) |  |
| 3 | 33 (22.5) | 15 (21.4) | 18 (23.4) |  |
| 4 | 19 (12.9) | 6 (8.6) | 13 (16.9) |  |
| 5 | 6 (4.1) | 3 (4.3) | 3 (3.9) |  |
| Diuretics | 28 (19.1) | 15 (21.4) | 13 (16.9) | 0.532 |
| CCBs | 95 (64.6) | 40 (57.1) | 55 (71.4) | 0.085 |
| ACEIs | 34 (23.1) | 18 (25.7) | 16 (20.8) | 0.558 |
| ARBS | 66 (44.9) | 30 (42.9) | 36 (46.8) | 0.740 |
| Beta-blockers | 57 (38.8) | 22 (31.43) | 35 (45.5) | 0.092 |
| Peripheral alpha-1 blockers | 27 (18.4) | 11 (15.7) | 16 (20.8) | 0.524 |
| Central acting alpha-2 agonists | 9 (6.1) | 4 (5.7) | 5 (6.5) | 1.000 |
| Direct vasodilators | 13 (8.8) | 10 (14.3) | 3 (3.9) | 0.040 |
| Evening drug administration  n (%) | 73 (49.7) | 34 (48.6) | 39 (50.7) | 0.869 |
| **Laboratory results** |  |  |  |  |
| Hemoglobin (g/L)* | 132 (119, 145) | 134 (123, 147) | 130 (118, 140) | 0.062 |
| Hematocrit (%) | 40.4 + 4.9 | 41.1 + 4.7 | 39.7 + 4.9 | 0.078 |
| RDW (%)* | 13.6 (12.9, 14.6) | 13.4 (12.7, 14.6) | 13.8 (13.0, 14.6) | 0.254 |
| MCV (fl)* | 88.5 (81.6, 91.4) | 87.8 (80.4, 91.4) | 49.1 (84.1, 91.3) | 0.515 |
| NLR* | 1.84 (1.42, 2.46) | 1.87 (1.37, 2.52) | 1.84 (1.42, 2.35) | 0.860 |
| PLR* | 121.82  (96.88, 159.05) | 131.01  (105.70, 160.09) | 111.26  (87.1, 155.34) | 0.058 |
| Fasting plasma glucose (mmol/L)* | 5.7 (5.2, 6.4) | 5.4 (5.0, 6.2) | 5.9 (5.4, 6.7) | 0.002 |
| HbA1c (%)* | 6.1 (5.7, 6.6) | 6.1 (5.9, 6.5) | 6.0 (5.6, 6.7) | 0.842 |
| Cholesterol (mmol/L) | 4.59 + 0.89 | 4.72 + 0.89 | 4.44 + 0.88 | 0.084 |
| Triglyceride (mmol/L)* | 1.20 (0.94, 1.72) | 1.26 (0.97, 1.90) | 1.15 (0.91, 1.55) | 0.180 |
| HDL-cholesterol (mmol/L) | 1.52 + 0.46 | 1.49 + 0.50 | 1.54 + 0.41 | 0.442 |
| LDL-cholesterol (mmol/L) | 2.47 + 0.81 | 2.58 + 0.85 | 2.38 + 0.77 | 0.147 |
| Uric acid (mmol/L) | 0.35 + 0.11 | 0.36 + 0.11 | 0.33 + 0.10 | 0.271 |
| Abnormal proteinuria n (%) | 47 (32.0) | 17 (24.3) | 30 (39.0) | 0.462 |
| Serum creatinine (μmol/L)* | 81.33  (65.42, 103.43) | 78.68  (62.76, 98.12) | 83.10  (68.95, 105.20) | 0.422 |
| eGFR (ml/min/1.73 m^2^) | 75.63 + 24.17 | 80.06 + 23.69 | 71.60 + 24.04 | 0.034 |

BMI = body mass index; CCBs = calcium channel blockers; ACEIs = angiotensin converting enzyme inhibitors; ARBs = angiotensin II receptor blockers; RDW = red blood cell distribution width; MCV = mean cell volume; NLR = neutrophil-lymphocyte ratio; PLR = platelet-lymphocyte ratio; HbA1c = hemoglobin A1c; HDL = high density lipoprotein; LDL = low density lipoprotein, eGFR = estimated glomerular filtration rate

*presented as median and 25^th^, 75^th^ percentile

**Supplementary Table 3:** **Hemodynamic data of treated hypertensive groups.**

| Blood pressure | Total  (n = 61) | Dippers  (n = 34) | Non-dippers  (n = 27) | P-value |
| --- | --- | --- | --- | --- |
| Average office SBP (mmHg) | 150.1 + 18.0 | 150.7 + 19.5 | 149.5 + 16.6 | 0.676 |
| Average office DBP (mmHg) | 83.9 + 12.3 | 87.9 +12.4 | 80.3 + 11.0 | <0.001 |
| 24hours average SBP (mmHg) | 130.4 + 14.7 | 131.2 + 14.3 | 129.7 + 15.1 | 0.544 |
| 24hourrs average DBP (mmHg) | 71.9 + 11.8 | 75.3 + 13.1 | 68.8 + 9.6 | <0.001 |
| Daytime SBP (mmHg) | 133.7 + 15.9 | 137.6 + 15.3 | 130.1 + 15.8 | 0.004 |
| Daytime DBP (mmHg) | 74.0 + 13.1 | 79.4 + 14.0 | 69.2 + 10.1 | <0.001 |
| Nighttime SBP (mmHg) | 123.7 + 17.3 | 117.1 + 13.8 | 129.8 + 18.0 | <0.001 |
| Nighttime DBP (mmHg) | 67.3 + 11.8 | 66.4 + 12.2 | 68.0 + 11.5 | 0.422 |

SBP = systolic blood pressure; DBP = diastolic blood pressure

**Supplementary Table 4: Demographic characteristics of untreated hypertensive patients.**

| **Characteristics** | **Total**  **(n = 61)** | **Dippers**  **(n = 34)** | **Non-dippers**  **(n = 27)** | **P-value** |
| --- | --- | --- | --- | --- |
| Age (years) | 51.5 + 16.7 | 47.4 + 17.7 | 56.7 + 14.2 | 0.289 |
| Male n (%) | 17 (27.9) | 11 (32.4) | 6 (22.2) | 0.408 |
| Body weight (kg) | 62.2 + 13.0 | 65.1 + 14.9 | 58.5 + 9.0 | 0.482 |
| BMI (kg/m^2^)* | 22.8 (20.3, 25.8) | 23.0 (21.3, 26.4) | 22.8 (19.0, 25.3) | 0.131 |
| Smoking n(%) | 1 (1.6) | 1 (2.9) | 0 (0) | 1.000 |
| **Co-morbidities n (%)** |  |  |  |  |
| Diabetes mellitus | 5 (8.2) | 2 (5.9) | 3 (11.1) | 0.647 |
| Dyslipidemia | 17 (27.9) | 9 (26.5) | 8 (29.6) | 1.000 |
| Obstructive sleep apnea | 1 (1.6) | 1 (2.9) | 0 (0) | 1.000 |
| Ischemic stroke | 4 (6.6) | 0 (0) | 4 (14.8) | 0.034 |
| Coronary artery disease | 1 (1.6) | 1 (2.9) | 0 (0) | 1.000 |
| Heart failure | 1 (1.6) | 1 (2.9) | 0 (0) | 1.000 |
| Chronic kidney disease | 4 (6.6) | 0 (0) | 4 (14.8) | 0.034 |
| **Laboratory results** |  |  |  |  |
| Hemoglobin (g/L)* | 133 (125, 144) | 137 (128, 150) | 129 (116, 140) | 0.043 |
| Hematocrit (%) | 41.2 + 4.5 | 42.4 + 4.1 | 39.6 + 4.6 | 0.015 |
| RDW (%)* | 13.4 (12.7, 14.4) | 13.3 (12.7, 14.4) | 13.5 (12.4, 14.8) | 0.722 |
| MCV (fl)* | 88.5 (82.4, 92.4) | 87.7 (82.4, 89.4) | 91.4 (82.2, 93.3) | 0.105 |
| NLR* | 1.71 (1.21, 2.42) | 1.51 (1.20, 2.16) | 1.88 (1.21, 2.46) | 0.416 |
| PLR* | 129.86  (98.04, 166.12) | 113.74  (94.29, 171.30) | 133.35  (117.3, 162.25) | 0.177 |
| Fasting plasma glucose (mmol/L)* | 5.3 (4.9, 5.7) | 5.2 (4.9, 5.5) | 5.4 (5.1, 6) | 0.176 |
| HbA1c (%)* | 5.7 (5.6, 6.0) | 5.7 (5.6, 6.0) | 5.9 (5.6, 6.2) | 0.388 |
| Cholesterol (mmol/L) | 5.04 + 0.88 | 5.08 + 0.86 | 4.99 + 0.93 | 0.682 |
| Triglyceride (mmol/L)* | 1.00 (0.79, 1.40) | 1.00 (0.71, 1.31) | 1.00 (0.79, 1.40) | 0.833 |
| HDL-cholesterol (mmol/L) | 1.57 + 0.41 | 1.57 + 0.41 | 1.56 + 0.42 | 0.964 |
| LDL-cholesterol (mmol/L) | 2.94 + 0.82 | 3.03 + 0.81 | 2.83 + 0.82 | 0.347 |
| Uric acid (mmol/L) | 0.33 + 0.10 | 0.35 + 0.08 | 0.32 + 0.11 | 0.270 |
| Abnormal proteinuria n (%) | 8 (13.1) | 3 (0.1) | 5 (0.2) | 1.000 |
| Serum creatinine (μmol/L)* | 68.95  (61, 79.56) | 72.49  (64.53, 83.10) | 65.42  (58.34, 77.79) | 0.193 |
| eGFR (ml/min/1.73 m^2^) | 91.27 + 20.77 | 94.22 + 18.32 | 87.55 + 23.32 | 0.216 |

BMI = body mass index; RDW = red blood cell distribution width; MCV = mean cell volume; NLR = neutrophil-lymphocyte ratio; PLR = platelet-lymphocyte ratio; HbA1c = hemoglobin A1c; HDL = high density lipoprotein; LDL = low density lipoprotein, eGFR = estimated glomerular filtration rate

*presented as median and 25^th^, 75^th^ percentile

**Supplementary Table 5: Hemodynamic data of untreated hypertensive groups.**

| Blood pressure | Total  (n = 61) | Dippers  (n = 34) | Non-dippers  (n = 27) | P-value |
| --- | --- | --- | --- | --- |
| Average office SBP (mmHg) | 140.5 + 12.9 | 141.5 + 9.5 | 139.3 + 16.3 | 0.519 |
| Average office DBP (mmHg) | 81.3 + 10.8 | 82.3 +11.2 | 80.1 + 10.3 | 0.428 |
| 24hours average SBP (mmHg) | 124.3 + 13.9 | 122.5 + 11.9 | 126.6 + 16.1 | 0.250 |
| 24hourrs average DBP (mmHg) | 70.7 + 8.6 | 70.2 + 8.4 | 71.2 + 8.9 | 0.649 |
| Daytime SBP (mmHg) | 128.6 + 14.1 | 128.6 + 12.4 | 128.6 + 16.3 | 0.995 |
| Daytime DBP (mmHg) | 73.6 + 9.2 | 74.6 + 9.3 | 72.4 + 9.1 | 0.369 |
| Nighttime SBP (mmHg) | 114.6 + 15.0 | 109.0 + 11.2 | 121.7 + 16.3 | <0.001 |
| Nighttime DBP (mmHg) | 64.3 + 9.4 | 60.6 + 8.2 | 68.9 + 8.8 | <0.001 |

SBP = systolic blood pressure; DBP = diastolic blood pressure

**Supplementary Table 6: Univariable logistic regression analysis for evaluation of the association between risk factors and non-dippers in treated hypertensive group.**

| Factors | Univariable analysis | |
| --- | --- | --- |
|  | **Odds ratio**  **(95% CI)** | **P-value** |
| Age | 1.038 (1.015 – 1.063) | 0.001 |
| Age > 65 years | 3.462 (1.746 – 6.866) | <0.001 |
| Male | 0.605 (0.312 – 1.175) | 0.138 |
| Body weight | 0.985 (0.961 – 1.010) | 0.240 |
| BMI | 1.001 (0.940 – 1.065) | 0.983 |
| Diabetes mellitus | 3.615 (1.643 – 7.953) | 0.001 |
| Dyslipidemia | 3.482 (1.624 – 7.466) | 0.001 |
| Obstructive sleep apnea | 0.900 (0.299 – 2.708) | 0.851 |
| Ischemic stroke | 2.873 (0.560 – 14.731) | 0.206 |
| Coronary artery disease | 1.887 (0.454 – 7.851) | 0.383 |
| Chronic kidney disease | 1.644 (0.751 – 3.601) | 0.214 |
| Diuretics | 0.745 (0.326 – 1.700) | 0.484 |
| CCBs | 1.875 (0.946 – 3.718) | 0.072 |
| ACEIs | 0.758 (0.351 – 1.634) | 0.479 |
| ARBs | 1.171 (0.610 – 2.246) | 0.635 |
| Beta-blockers | 1.818 (0.926 – 3.572) | 0.083 |
| Peripheral alpha-1 blockers | 1.407 (0.603 – 3.282) | 0.430 |
| Central acting alpha-2 agonists | 1.146 (0.295 – 4.449) | 0.844 |
| Direct vasodilators | 0.243 (0.064 – 0.924) | 0.038 |
| Evening drug administration | 1.087 (0.569 – 2.076) | 0.801 |
| Average office SBP | 0.996 (0.978 – 1.014) | 0.674 |
| Average office DBP | 0.945 (0.917 – 0.975) | <0.001 |
| Hemoglobin | 0.831 (0.684 – 1.009) | 0.062 |
| Hematocrit | 0.940 (0.877 – 1.008) | 0.081 |
| RDW | 0.974 (0.807 – 1.175) | 0.782 |
| MCV | 1.014 (0.980 – 1.049) | 0.424 |
| NLR | 1.071 (0.836 – 1.373) | 0.587 |
| PLR | 1.001 (0.998 – 1.004) | 0.454 |
| Fasting plasma glucose | 1.022 (1.004 – 1.041) | 0.019 |
| Fasting plasma glucose > 5.6 mmol/L | 3.122 (1.586 – 6.146) | 0.001 |
| HbA1c | 1.289 (0.857 – 1.938) | 0.222 |
| Cholesterol | 0.992 (0.982 -1.001) | 0.086 |
| Triglyceride | 0.994 (0.988 – 1.000) | 0.068 |
| HDL-cholesterol | 1.007 (0.989 – 1.026) | 0.440 |
| LDL-cholesterol | 0.992 (0.982 – 1.003) | 0.148 |
| Uric acid | 0.888 (0.719 – 1.097) | 0.270 |
| Abnormal proteinuria | 1.352 (0.647 – 2.823) | 0.423 |
| Serum creatinine | 1.116 (0.548 – 2.274) | 0.762 |
| eGFR | 0.985 (0.971 – 0.999) | 0.036 |

BMI = body mass index; CCBs = calcium channel blockers; ACEIs = angiotensin converting enzyme inhibitors; ARBs = angiotensin II receptor blockers; RDW = red blood cell distribution width; MCV = mean cell volume; NLR = neutrophil-lymphocyte ratio; PLR = platelet-lymphocyte ratio; HbA1c = hemoglobin A1c; HDL = high density lipoprotein; LDL = low density lipoprotein, eGFR = estimated glomerular filtration rate

**Supplementary Table 7: Univariable logistic regression analysis for evaluation of the association between risk factors and non-dippers in untreated hypertensive group.**

| Factors | Univariable analysis | |
| --- | --- | --- |
|  | **Odds ratio**  **(95% CI)** | **P-value** |
| Age | 1.037 (1.003 – 1.073) | 0.034 |
| Age > 65 years | 2.745 (0.845 – 8.915) | 0.093 |
| Male | 0.567 (0.188 – 1.901) | 0.383 |
| Body weight | 0.954 (0.910 – 1.001) | 0.056 |
| BMI | 0.912 (0.811 – 1.025) | 0.122 |
| Diabetes mellitus | 2.000 (0.310 – 12.926) | 0.467 |
| Dyslipidemia | 1.170 (0.380 – 3.598) | 0.785 |
| Average office SBP | 0.987 (0.947 – 1.028) | 0.514 |
| Average office DBP | 0.981 (0.935 – 1.029) | 0.422 |
| Hemoglobin | 0.656 (0.456 – 0.942) | 0.023 |
| Hematocrit | 0.856 (0.750 – 0.978) | 0.022 |
| RDW | 1.109 (0.787 – 1.561) | 0.555 |
| MCV | 1.009 (0.952 – 1.069) | 0.762 |
| NLR | 1.151 (0.738 – 1.795) | 0.536 |
| PLR | 1.005 (0.995 – 1.014) | 0.341 |
| Fasting plasma glucose | 1.022 (0.987 – 1.058) | 0.224 |
| Fasting plasma glucose > 5.6 mmol/L | 1.838 (0.603 – 5.608) | 0.285 |
| HbA1c | 2.106 (0.542 – 8.189) | 0.282 |
| Cholesterol | 0.997 (0.982 -1.012) | 0.676 |
| Triglyceride | 1.004 (0.993 – 1.015) | 0.509 |
| HDL-cholesterol | 0.999 (0.968 – 1.032) | 0.963 |
| LDL-cholesterol | 0.992 (0.976 – 1.009) | 0.342 |
| Uric acid | 0.796 (0.532 – 1.192) | 0.268 |
| Abnormal proteinuria | 1.364 (0.286 – 6.496) | 0.697 |
| Serum creatinine | 0.954 (0.085 – 10.692) | 0.969 |
| eGFR | 0.984 (0.960 – 1.009) | 0.216 |

BMI = body mass index; CCBs = calcium channel blockers; ACEIs = angiotensin converting enzyme inhibitors; ARBs = angiotensin II receptor blockers; RDW = red blood cell distribution width; MCV = mean cell volume; NLR = neutrophil-lymphocyte ratio; PLR = platelet-lymphocyte ratio; HbA1c = hemoglobin A1c; HDL = high density lipoprotein; LDL = low density lipoprotein, eGFR = estimated glomerular filtration rate
