# Supplementary figures and images for: Clinical risk factors and predictive score for the non-dipper profile in hypertensive patients: a case-control study
Source: Clin Hypertens. 2021 Nov 15;27:22. doi: 10.1186/s40885-021-00180-4 (PMC8591836; doi:10.1186/s40885-021-00180-4)

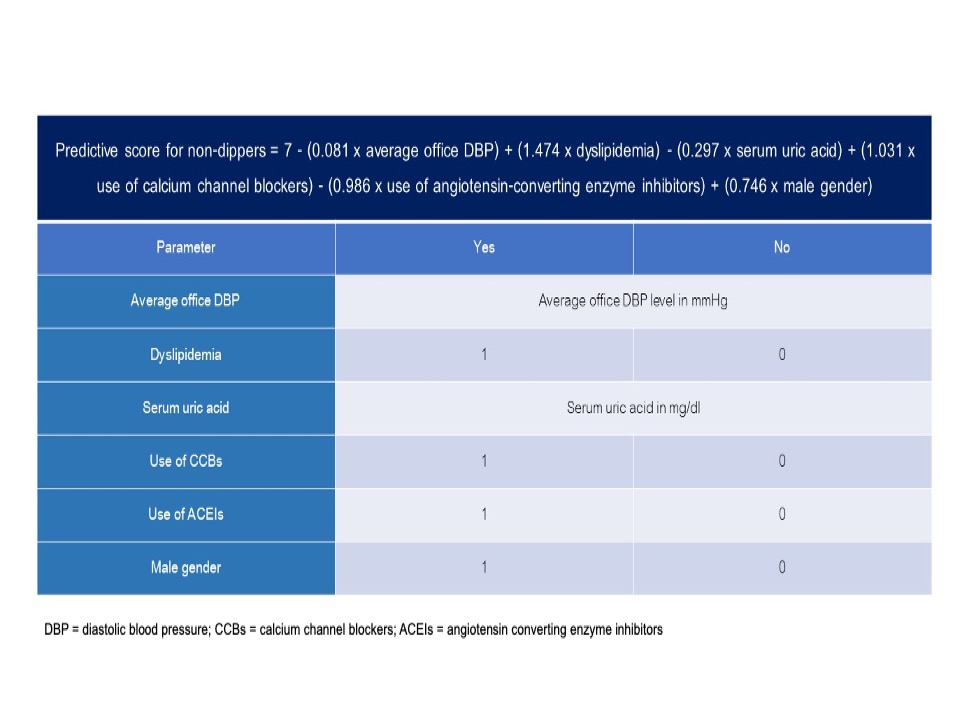

Supplement: Supplementary file 2 — Additional file 2: Supplementary Figure 1. Predictive score model for non-dippers in treated hypertensive patients. [file 40885_2021_180_MOESM2_ESM.jpeg]

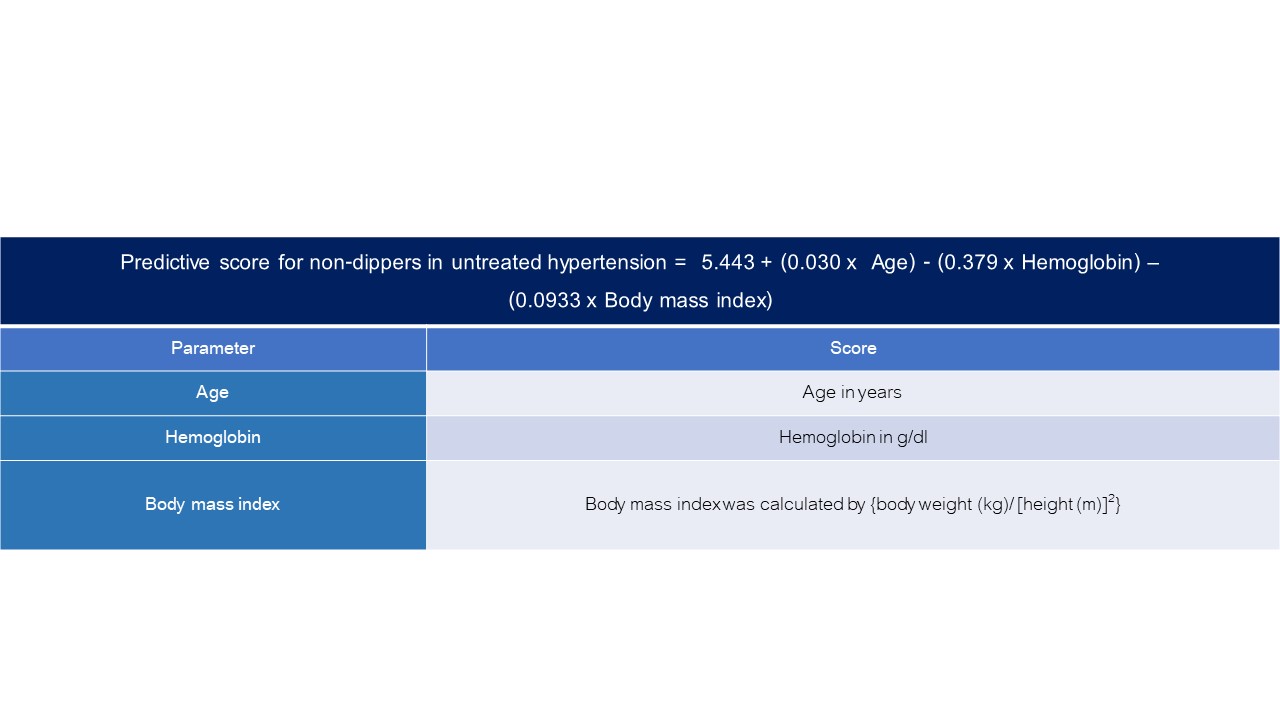

Supplement: Supplementary file 3 — Additional file 3: Supplementary Figure 2. Predictive score model for non-dippers in untreated hypertensive patients. [file 40885_2021_180_MOESM3_ESM.jpg]
